# CaMKII and MEK1/2 inhibition time-dependently modify inflammatory signaling in rat cerebral arteries during organ culture

**DOI:** 10.1186/1742-2094-11-90

**Published:** 2014-05-16

**Authors:** Roya Waldsee, Sajedeh Eftekhari, Hilda Ahnstedt, Leif E Johnson, Lars Edvinsson

**Affiliations:** 1 Department of Clinical Sciences, Division of Experimental Vascular Research, Lund University, Sölvegatan 17, SE-221 84 Lund, Sweden; 2 Department of Ophthalmology, Division of Clinical Science, Lund University, Klinikgatan 26, SE- 221 85 Lund, Sweden

**Keywords:** ERK1/2, CaMKII, Organ culture, Inflammation, JNK, p38

## Abstract

**Background:**

Cerebral ischemia induces transcriptional upregulation of inflammatory genes in the brain parenchyma and in cerebral arteries, thereby contributing to the infarct development. The present study was designed to evaluate the involvement of calcium-calmodulin-dependent protein kinase (CaMKII) II and extracellular signal-regulated kinase1/2 (ERK1/2) on inflammatory mediators in rat cerebral arteries using organ culture as a method for inducing ischemic-like vascular wall changes.

**Methods:**

Rat basilar arteries were cultured in serum-free medium for 0, 3, 6 or 24 hours in the presence or absence of the CaMKII inhibitor KN93 or the MEK1/2 inhibitor U0126. Protein expression of activated CaMKII, ERK1/2, and inflammatory-associated protein kinases and mediators were examined with western blot and immunohistochemistry. Caspase-3 mRNA levels in basilar arteries were studied with real-time PCR.

**Results:**

Western blot evaluation showed that organ culture induced a significant increase in phosphorylated ERK1/2 at 3, 6 and 24 hours, while CaMKII was found to be already activated in fresh non-incubated arteries and to decrease with incubation time. The addition of U0126 or KN93 decreased levels of phosphorylated c-Jun N-terminal kinase and p-p38, as evaluated by immunohistochemistry. KN93 affected the increase in caspase-3 mRNA expression only when given at the start of incubation, while U0126 had an inhibitory effect when given up to six hours later. Tumor necrosis factor receptor 1 was elevated after organ culture. This inflammatory marker was reduced by both of the two different protein kinase inhibitors.

**Conclusions:**

The novel findings of the present study are that the cross-talk between the two protein kinases and the inhibition of CaMKII or MEK1/2 in a time-dependent manner attenuates inflammatory-associated protein kinases and mediators, suggesting that they play a role in cerebrovascular inflammation.

## Introduction

Cerebral ischemia is well-known to be associated with inflammation [1], and its inhibition may result in reduced infarct volume [2,3]. Experimental middle cerebral artery occlusion (MCAO) and subarachnoid hemorrhage (SAH) have been shown to be associated with inflammation in the walls of cerebral arteries and in the microvasculature [4-7]. We have developed a method of isolated cerebral artery culture which simulates some aspects of cerebrovascular inflammation [8,9]. A multitude of stimuli have been identified that induce inflammation and apoptosis in vascular smooth muscle cells (VSMCs) including nitric oxide donors [10], serum starvation [11], tumor necrosis factor alpha (TNF-α), and interleukin-1 (IL-1) [12]. Organ culture in serum-free medium results in inflammation and apoptosis in isolated vessel segments via activation of different intracellular signals, such as induction of caspase cascades or upregulation of Fas ligand expression [13].

The mitogen-activated protein kinases (MAPK) are a group of serine/threonine kinases that play an important role in intracellular signaling [14]. There are three major MAPK pathways: MAPK kinase/extracellular signal-regulated kinase1/2 (MEK/ERK1/2), p38, and c-Jun N-terminal kinase (JNK). Cerebral ischemia and organ culture induce early activation of ERK1/2 and somewhat delay activation of p38 and JNK, thus demonstrating different time frames of activity after cerebral ischemia [15,16]. This in turn activates transcription factor nuclear factor kappa B (NF-κB) and c-Jun, and the subsequent expression of inflammatory genes in cerebral arteries [7,17].

Calcium/calmodulin-dependent protein kinase II (CaMKII) is a multifunctional serine/threonine kinase that affects different biological processes including gene expression, cell-cycle control, and hormone production [18]. In addition, CaMKII involvement has been shown in neuronal apoptosis [19]. Previous organ culture studies have demonstrated activation of MAPK kinases (MEK/ERK1/2) in middle cerebral arteries (MCAs) [20,21] and CaMKII in basilar arteries [22] in parallel to endothelin receptor B (ET_B_) receptor upregulation. These studies revealed a time-dependent effect of MEK/ERK1/2 and CaMKII inhibitors on ET_B_ receptor upregulation at protein and functional levels.

The present study analyzes the time course of stress signaling in response to organ culture by characterizing the phosphorylation of CaMKII, ERK1/2, JNK, and p38, and the levels of caspase-3, an executor of apoptosis. In addition, we studied TNF-α-receptor 1 (TNFR1) since TNF-α initiates inflammation in many cell types [23,24] via its receptor and has a role in cerebrovascular inflammation [5]. We hypothesize that organ culture-induced inflammation may share some features with the *in vivo* situation following stroke, and therefore evaluated U0126 and KN93, specific inhibitors of MEK1/2 and of CaMKII, respectively, which have demonstrated beneficial effects *in vivo*[8].

## Methods

Male Sprague-Dawley rats (Scanbur, Stockholm, Sweden) weighing 250 - 300 g were housed under controlled conditions and used for the study. The Animal Ethics Committee, Lund, Sweden, approved the experiments (M217-03, M161-07).

### Tissue preparation

**Figure 1:**
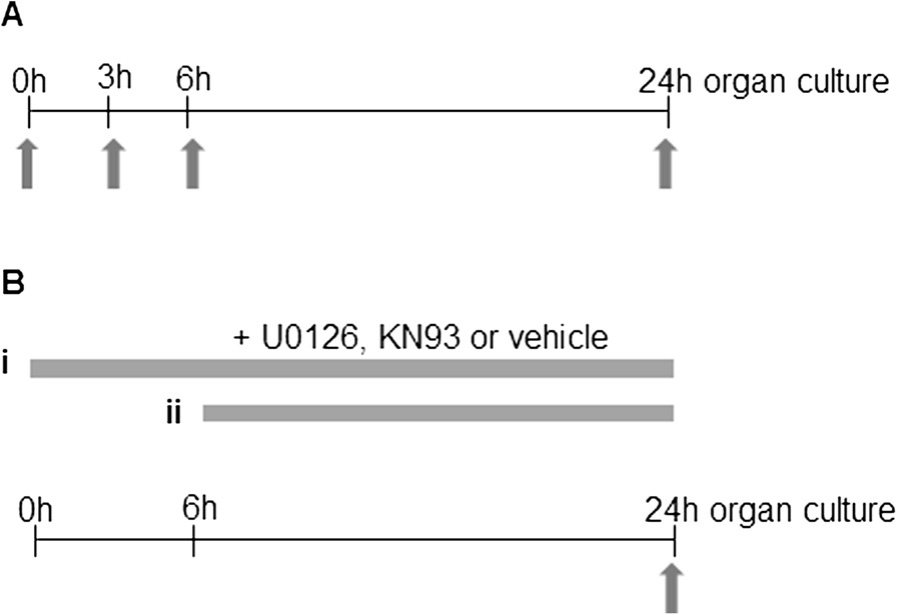
**Experimental design.** Rat basilar arteries were incubated according to **(A)** time study and **(B)** treatment study. Inhibitors or vehicle was administered at 0 hours (i) or at 6 hours (ii). Arrows indicate end points where arteries were collected for further studies.

The animals (total number used, n = 113) were sacrificed by CO_2_ followed by decapitation. The basilar arteries were isolated and placed in a cold bicarbonate buffer solution with the following composition (in mM): NaCl 119, NaHCO_3_15, KCl 4.6, NaH_2_PO_4_ 1.2, MgCl_2_ 1.2, CaCl_2_1.2, and glucose5.5. The arteries were incubated in Dulbecco’s modified Eagle’s medium (DMEM; Gibco, Life Technologies Europe BV, Stockholm, Sweden) supplemented with penicillin (100 U/mL), streptomycin (100 μg/ml), and amphotericin B (0.25 μg/ml) at 37°C in humidified 5% CO_2_ and air. The experimental design is depicted in Figure 1. For the time study, vessels were incubated in DMEM for 0, 3, 6 or 24 hours where 0 hours was considered as the control (Figure 1A). The CaMKII inhibitor KN93 (10^−5^ M) or the MEK1/2 inhibitor U0126 (10^−5^ M) were added at 0 or 6 hours after initiating the incubation (Figure 1B). Artery segments incubated with vehicle (equal volume of dimethyl sulfoxide (DMSO)) were used as the control in the treatment study.

### Tissue lysis and protein content determination

After incubation, the arteries were collected (total number of rats used n = 48) and homogenized in denaturing cell extraction buffer (Life Technologies Europe BV) supplemented with protease and phosphatase inhibitors (Sigma, St Louis, Missouri, United States). After 20 minutes of incubation on ice, homogenates were centrifuged at 13000 *g* for 10 minutes at 4°C and the supernatant was collected. Total protein concentration was determined using a Bio-Rad protein assay dye (Hercules, California, United States) and measurement of the absorbance at 595 nm on an Infinite M200 micro plate reader (Tecan, Männedorf, Switzerland) was obtained.

### Western blot analysis

Lysates were dissolved in a Laemmli sample buffer (Bio-Rad) supplemented with 2-mercaptoethanol (Bio-Rad) and boiled for 4 minutes at 95°C. Equal amounts of proteins (40 to 50 μg/lane) were loaded on a 4 to 15% linear gradient Trizma hydrochloride (Tris- HCl) gel (Bio-Rad) and separated by SDS-polyacrylamide gel electrophoresis. Molecular weight markers (Fisher Scientific, Bio-Rad) were loaded onto each gel for protein band identification. After separation, proteins were transferred onto a nitrocellulose (Bio-Rad) or polyvinylidene fluoride (Life Technologies) membrane. Subsequently, the membrane was blocked with 5% non-fat milk in Tween- Trizma Buffered Saline (T-TBS, pH 7.6) for 1 hour at room temperature, followed by three 5 minute washes with T-TBS. For detection of phosphorylated CaMKII, 1% non-fat milk and 1% bovine serum albumin (BSA) in T-TBS supplemented with protease and phosphatase inhibitors (Sigma) was used as a blocking solution. Membranes were then incubated with one of the following primary antibodies: mouse monoclonal anti-CaMKII phospho-specific (1:1000; Santa Cruz Biotechnology, Santa Cruz, California, United States), rabbit monoclonal anti-CaMKII (1:20 000; Abcam, Cambridge, United Kingdom), rabbit polyclonal anti-ERK1/2 phospho-specific (1:1000; Cell Signaling Technology, Beverly, Massachusetts, United States), mouse anti-phosphorylated-JNK (1:750, Santa Cruz Biotechnology), mouse anti-alpha tubulin (1:60000, Abcam) and mouse monoclonal anti-ERK1/2 (1:2000; Cell Signaling) overnight at 4°C, followed by three 5 minute washes with T-TBS. Subsequently, the membranes were incubated with the appropriate horseradish peroxidase conjugated secondary antibodies for 1 hour at room temperature, followed by four 5 minute washes with T-TBS and one 5 minute wash with TBS. The membranes were developed using a Supersignal West Dura kit (Pierce, Rockford, Illinois, United States) or a Western chemiluminescent horseradish peroxidase (HRP) solution (Millipore Billerica, MA, USA) and visualized using a luminescence image analyzer. For reprobing, blots were stripped by incubating the membranes for 45 minutes in stripping buffer (0.0625 M Tris-HCl containing 2% SDS and 0.007% 2-mercaptoethanol, pH 6.8), blocked again for 1 hour and treated as described above with new primary antibodies. Protein band densities were quantified using the Image J software (National Institutes of Health, Bethesda, Maryland, USA) or Image Lab 5.1 software (Bio Rad). The optical density values are presented as absolute ratios of the phosphorylated levels of CaMKII and ERK1/2 to the total levels, and for phosphorylated JNK the optical density values show the inhibitor-treated samples as a percent of the control value.

### Molecular biology

Basilar artery segments (total number of rats used n = 40) were incubated in DMEM for 0, 3, 6, or 24 hours for time-dependent evaluation of caspase-3 during organ culture. To evaluate the role of CaMKII or MEK/ERK1/2 in caspase-3 activation, basilar artery segments were cultured for 24 hours in the presence or absence of KN93 (10^−5^ M) or U0126 (10^−5^ M) added at 0 or 6 hours after initiating incubation (Figure 1B). The concentrations have been evaluated previously [21,22]. The vessel segments were immediately snap-frozen and stored at -80°C until use. Total cellular RNA was extracted using a FastRNA Pro Green kit (Qbiogene, Illkirsh, France) for 60 seconds in the FastPrep FP120 instrument (Qbiogene) following the manufacturer’s instructions. First strand cDNA was synthesized from 200 ng total RNA in a 40 μl reaction volume using random hexamers as primers. Quantitative real-time PCR (qPCR) was performed in a GeneAmp 5700 Sequence Detection System using the GeneAmp SYBR® Green kit (Perkin-Elmer, Applied Biosystems, Foster City, California, United States) with the cDNA synthesized above as the template in a 28 μl reaction volume. A sample without the template served as the control. Specific primers were designed as caspase-3; 5’-AATTCAAGGGACGGGTCATG-3’ as the forward and 5’-GCTTGTGCGCGTACAGTTTC-3’ as the reverse [25]. The housekeeping gene was elongation factor-1 (EF-1) with 5’-GCAAGCCCATGTGTGTTGAA-3’as the forward and 5’-TGATGACACCCACAGCAACTG-3’ as the reverse. In previous studies, we examined and compared EF-1, β-actin, and glyceraldehyde 3-phosphate dehydrogenase (GAPDH) as the housekeeping genes and verified that EF-1 is stable during the organ culture procedure [7].

### Immunohistochemistry

Basilar artery segments (total number of rats n = 25) were cultured for 24 hours with KN93 or U0126 administered at 0 or 6 hours after initiating incubation. Vessels incubated for 24 hours without inhibitor served as the controls. Subsequently, the basilar artery segments were embedded in Tissue TEK (Histolab, Västra Frölunda, Sweden), frozen at -80°C, and cryosectioned (10 μm, Cryo-star HM 560 M Thermo Scientific, Microm, Waltham, MA USA). The sections were fixed for 10 minutes in ice-cold acetone, followed by rehydration in phosphate buffered-saline (PBS, pH 7.2) containing 0.25% Triton X-100 (PBST) for three times at 5 minutes each, and incubated with blocking solution containing PBS and 5% normal donkey serum for 1 hour. After blocking, the sections were incubated overnight at 4°C with one of the following primary antibodies: rabbit anti-CaMKII (1:100; Abcam, Cambridge, United Kingdom), mouse anti-phospho-JNK (1:100, Santa Cruz Biotechnology), rabbit anti-phospho-p38 (1:50, Cell Signaling Technology) or rabbit anti TNF-α receptor I (1:1500, Abcam). The primary antibodies were diluted in PBST, containing 1% BSA and 3% normal donkey serum. After incubation with the primary antibodies, sections were washed three times (15 minutes each wash) in PBST and incubated with secondary antibody Cy™^2^-conjugated donkey anti-rabbit (1:200, Jackson ImmunoResearch, West Grove, Pennsylvania, United States) or donkey Texas-Red anti-mouse (1:100, Jackson ImmunoResearch) diluted in PBST and 1% BSA, for 1 hour at room temperature. The sections were subsequently washed three times in PBST for 15 minutes each wash, thereafter mounted with Crystal mounting medium (Sigma). Omission of primary antibodies served as negative controls for all antibodies. Sections were examined and images were obtained using an epifluorescence microscope (Olympus Microscope BX 60, Tokyo, Japan) at the appropriate wavelengths. Fluorescence intensity measurements were made in the media layer of the vessels with the use of the software program Image J (National Institutes of Health, Bethesda, Maryland, USA). For measurement analysis, there were five rats per group and between four and six sections from each rat were evaluated. The fluorescence intensity was measured in four areas in each section and the mean value of the intensity per measured area was used. The immunoreactivity of the individual receptors was visualized with the same microscope settings during the same day for all groups.

### Statistics

Results are given as mean ± SEM, and n refers to the number of rats. There were n = 3 samples per group in the western blot experiments (the basilar arteries from three rats were pooled to constitute on sample). In the immunohistochemistry experiments there were n = 5 rats in each group, while there were between four and six rats in the real-time PCR experiments. Kruskal-Wallis nonparametric test with Dunn’s *post-hoc* test was used for all statistical analyses. The level of significance was set to *P* <0.05.

## Results

### Activation of ERK1/2 and CaMKII during organ culture

**Figure 2:**
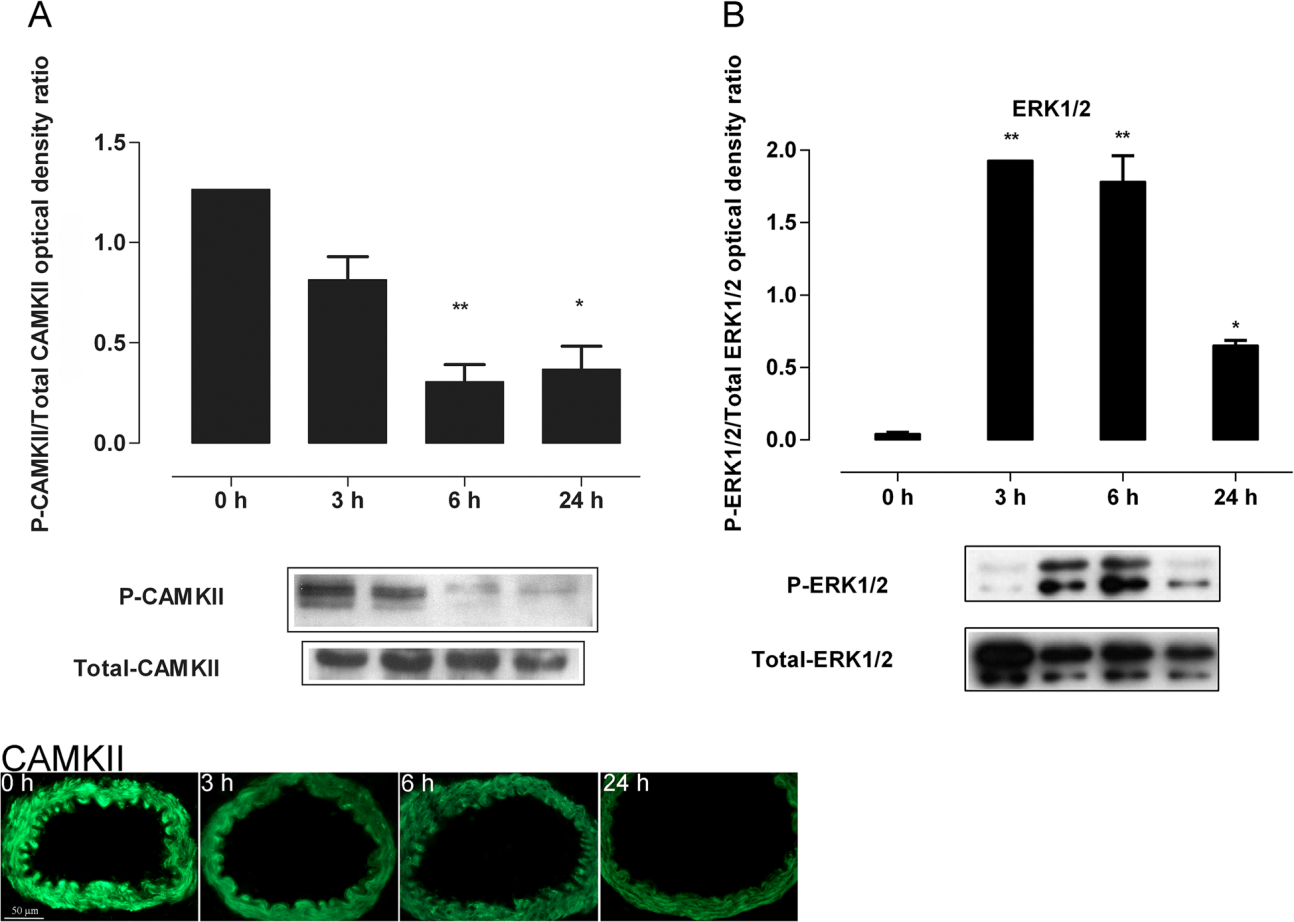
**Basilar artery segments were incubated for 0, 3, 6, or 24 hours.** Phosphorylated and total level of CaMKII **(A)** were measured by western blot and detected by immunofluorescence. **(B)** ERK1/2 was measured by western blot. Phosphorylated CaMKII and ERK1/2 were compared to the total levels of the kinases. Significant differences were obtained between fresh (0 hours) and incubated arteries for western blot. Data are shown as mean ± SEM, n = 5. *P <0.05. Extracellular signal regulated kinase 1/2 (ERK1/2), calcium calmodulin-dependent protein kinase II (CAMKII).

Time-dependent phosphorylation of ERK1/2 and CaMKII was evaluated by western blot. High levels of activated CaMKII were found in freshly isolated and non-incubated arteries (Figure 2A) in accordance with an earlier study [22]. The p-CaMKII expression decreased with incubation time to a significant difference at 6 and 24 hours (*P* <0.05). Immunofluorescence results showed similar results, CAMKII immunoreactivity was high at 0 hours and decreased during the time of organ culture (Figure 2A). In contrast, ERK1/2 was strongly activated at 3 hours after initiating incubation and remained elevated for up to 24 hours compared to 0 hours (*P* <0.05, Figure 2B). These results show apparent differences in p-ERK1/2 and p-CaMKII levels at 0 and 6 hours of incubation. Therefore, MEK1/2 or CaMKII inhibitors were added at these time points to investigate the time-dependent effect of these inhibitors on inflammatory mediators.

### Caspase-3 during organ culture

**Figure 3:**
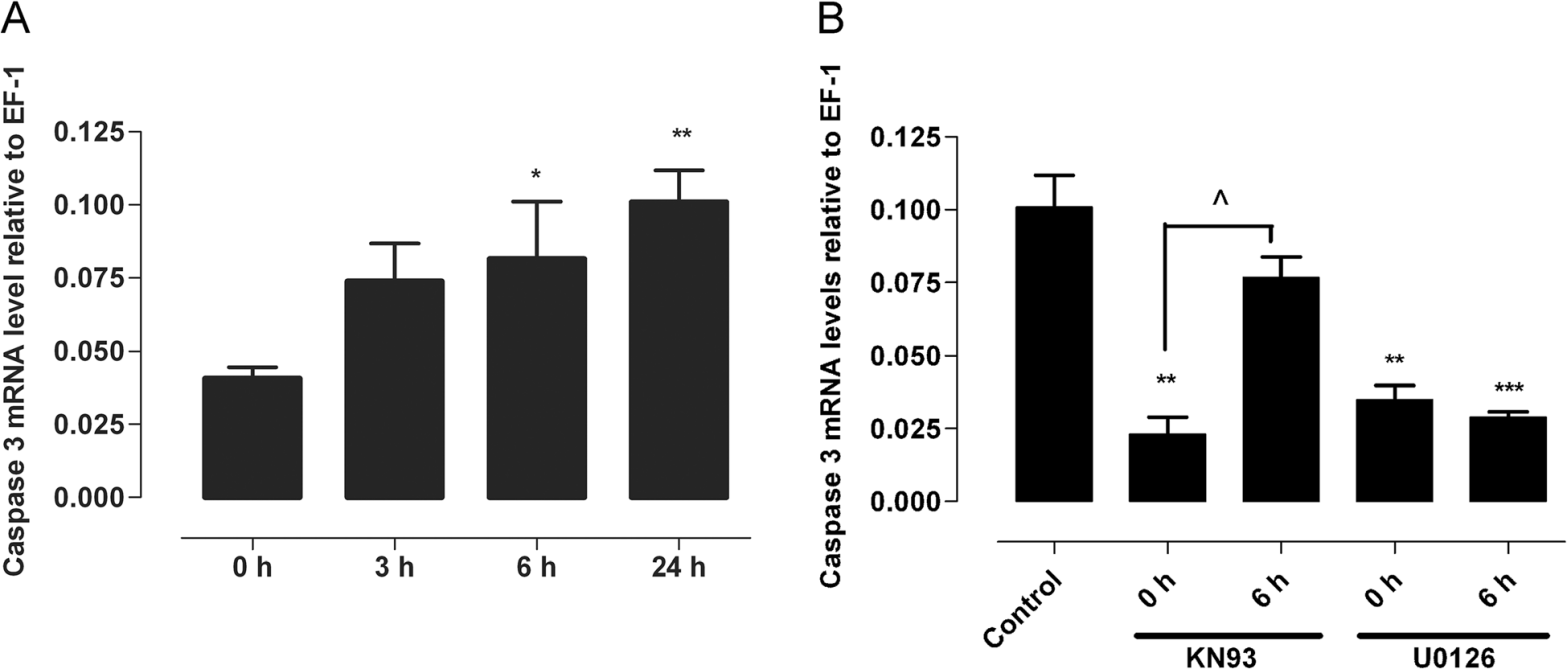
**Relative caspase-3 mRNA levels in incubated arteries. (A)** evaluated in arteries incubated for 0 (fresh), 3, 6 or 24 hours in serum-free DMEM (0 hours was used as control) and **(B)** in arteries incubated for 24 hours with or without CaMKII inhibitor (KN93, 10^−5^ M) or MEK1/2 inhibitor (U0126, 10^−5^ M) added at 0 or 6 hours after initiating incubation (24-hour-incubated arteries with vehicle were used as control). Data are presented as mean ± SEM relative to (elongation factor-1) EF-1, n = 4–6. *P <0.05 **P <0.01, ***P <0.001. ^*P* <0.05 represents significant difference between 0 and 6 hours in arteries incubated with KN93. Dulbecco’s modified eagle’s medium (DMEM), calcium calmodulin-dependent protein kinase II (CAMKII).

Caspase-3 mRNA level was evaluated at 0, 3, 6 and 24 hours after organ culture by real-time PCR [25]. The mRNA level of caspase-3 increased in a time-dependent manner and was significantly elevated at 6 and 24 hours (*P* <0.05 and *P* <0.01, Figure 3A). Furthermore, the effects of the inhibitors U0126 and KN93 on caspase-3 mRNA levels were evaluated. Figure 3B demonstrates a significant (*P* <0.01) reduction in caspase-3 mRNA levels in arteries incubated with KN93 only if it was added at the start of incubation (0 hours). KN93 caused a significant reduction of caspase-3 mRNA levels if it was added at 0 hours, but did not have a significant effect when given at 6 hours after initiation of incubation (*P* <0.05). U0126 on the other hand, added at 0 or 6 hours after initiating incubation, significantly decreased caspase-3 mRNA levels (*P* <0.01 and *P* <0.001, respectively). These results show that U0126 has an inhibitory effect when added at either 0 or 6 hours after initiating incubation.

### Effects of KN93 and U0126 on p-JNK and p-p38

**Figure 4:**
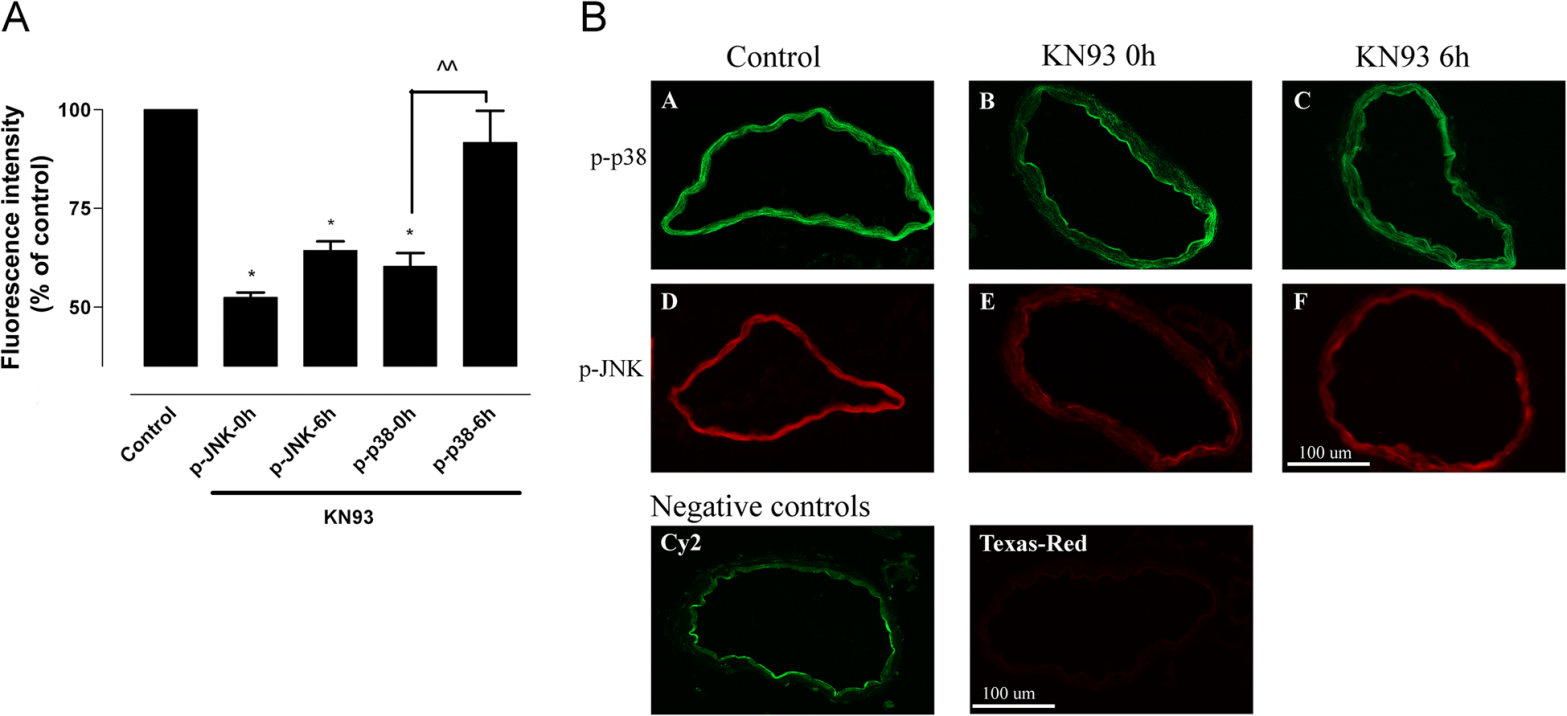
**Effect of the CAMKII inhibitor on p-p38 and p-JNK levels in incubated arteries (A) Fluorescence intensity and (B) expression of p-p38 and p-JNK in the vascular smooth muscle cell layer of cerebral arteries incubated for 24 hours in presence or absence of the CaMKII inhibitor KN93 added at 0 or 6 hours after initiating incubation.** (A, D) Arteries incubated in the presence of vehicle for 24 hours were used as the control and treatments compared to this level of expression (set as 100%). (B, E) KN93 added at 0 hours, (C, F) KN93 added at 6 hours. Data are presented as mean ± SEM relative to control, n = 5, *P <0.05. ^^*P* <0.01 represents significant difference in p-p38 protein level between 0 and 6 hours. Negative controls (omission of primary antibody) for Cy^2^ -conjugated donkey anti-rabbit and Texas-Red-conjugated donkey anti-mouse. Calcium calmodulin-dependent protein kinase II (CAMKII), phosphorylated c-Jun N-terminal kinase (p-JNK).

Protein analysis by immunohistochemistry revealed that KN93 added at 0 or 6 hours after initiating incubation significantly decreased the level of phosphorylated c-Jun N-terminal kinase (p-JNK) compared to the control (24-hours incubation in the presence of vehicle) (*P* <0.05, Figure 4). Western blot analysis showed that p-JNK decreased by 16% in KN93-treated samples (0 hours) as compared to untreated controls, but this difference was not statistically significant (data not shown). Phosphorylated p38 was significantly reduced by KN93 given at 0 hours (*P* <0.05) but not at 6 hours (*P* >0.05, Figure 4). Thus, KN93 had a differential inhibitory effect of p-JNK and p-p38.

**Figure 5:**
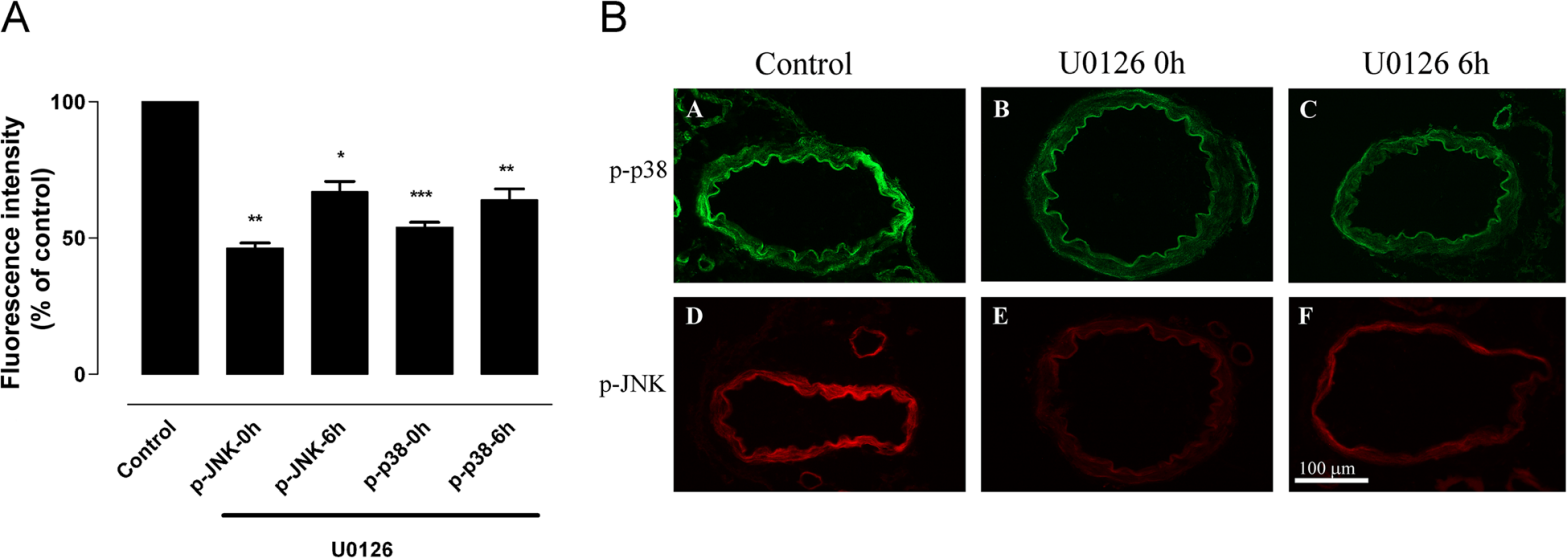
**Effect of the MEK1/2 inhibitor on p-p38 and p-JNK levels in incubated arteries (A) Fluorescence intensity and (B) expression of p-p38 and p-JNK in the vascular smooth muscle cell layer of cerebral arteries incubated for 24 hours in presence or absence of the MEK1/2 inhibitor U0126 added at 0 or 6 hours after initiating incubation.** (A, D) Arteries incubated for 24 hours in presence of vehicle were used as control. (B, E) U0126 added at 0 hours, (C, F) U0126 added at 6 hours. Data are presented as mean ± SEM relative to control. n = 5 *P <0.05, **P <0.01, ***P <0.001. Calcium calmodulin-dependent protein kinase II (CAMKII), phosphorylated c-Jun N-terminal kinase (p-JNK).

MEK1/2 inhibition had similar effects on p-JNK when added at 0 hours (*P* <0.01) or at 6 hours (*P* <0.05) after start of incubation (Figure 5). Western blot analysis showed that p-JNK decreased by 23% in KN93-treated samples (0 hours) as compared to untreated controls, but this difference was not statistically significant (data not shown). U0126 also decreased p-p38 levels at both time points (at 0 hours *P* <0.001 and at 6 hours *P* <0.01, Figure 5). There was no significant difference in the inhibitory effect of U0126 when given at 0 or 6 hours. The results show that both inhibitors affect the activation of p38 and JNK at 24 hours of incubation, but only U0126 had an inhibitory effect on p-p38 when given at 6 hours after initiating incubation.

### Effect of KN93 and U0126 on TNFR1 expression

**Figure 6:**
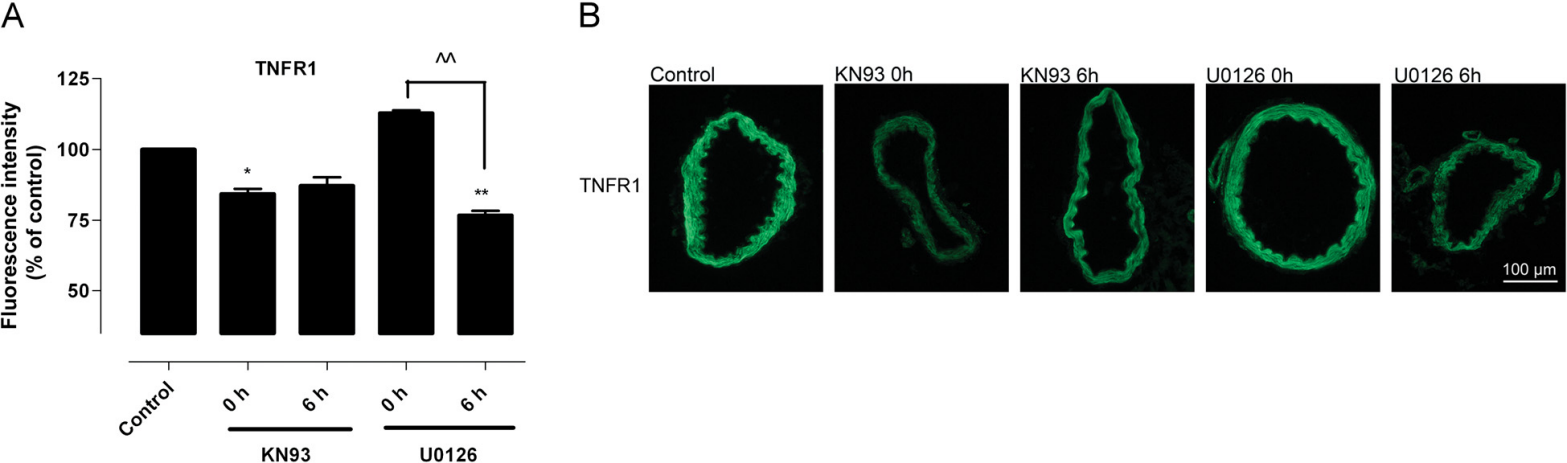
**Effect of the MEK1/2 inhibitor or the CAMKII inhibitor on TNFR1 levels in incubated arteries (A) Fluorescence intensity and (B) expression of TNFR1 in the vascular smooth muscle cell layer of cerebral arteries incubated for 24 hours in presence or absence of the MEK1/2 inhibitor U0126 or the CAMKII inhibitor KN93, added at 0 or 6 hours after initiating incubation.** Data are presented as mean ± SEM relative to 24 hours incubation in presence of vehicle (control). n = 5. *P <0.05, **P <0.01. ^^*P* <0.01 represents significant difference in TNFR1 protein level between 0 and 6 hours in arteries treated with U0126. Calcium calmodulin-dependent protein kinase II (CAMKII), tumor necrosis factor receptor 1 (TNFR1).

We also investigated whether inhibition of MEK1/2 or CaMKII had any effect on expression of the putative initiator of inflammatory signaling, the TNF-α receptor. We therefore studied TNFR1 protein expression that is one of the starting signals in inflammatory activity [23,24]. Previous work has demonstrated that organ culture elicits an enhanced expression of TNF-α and TNFR1 both after organ culture and during *in vivo* stroke [5]. The present results revealed that incubation with U0126 or KN93 lowered the protein level of TNFR1 at 24 hours of incubation compared to control (set to 100%) (Figure 6). KN93 had a significant effect (*P* <0.05) only when added at 0 hours of incubation, while U0126 significantly reduced TNFR1 protein expression only when added at 6 hours of incubation (*P* <0.01). The results demonstrate time-dependent differences in the inhibitory effect of U0126 and KN93.

## Discussion

CaMKII and MEK1/2 have been shown to be involved in cerebrovascular receptor upregulation after cerebral ischemia [8,22]. The exact mechanisms involved in vascular inflammation are, however, poorly understood [1]. Earlier studies have revealed that cerebral ischemia and organ culture in particular activates intracellular signaling kinases including CaMKII [22], MEK/ERK1/2, JNK, p38, inflammatory cytokines, and metalloproteinases in cerebral arteries [15,17,26]. Activation of MEK/ERK1/2 occurs in parallel with ET_B_ receptor upregulation after organ culture [21,22], in SAH [27] and MCAO [28]. In contrast, CaMKII shows a lower degree of activation (p-CaMKII expression) and KN93 has an effect only when the inhibitor is given early or in conjunction with the cerebral ischemia [29]. The inhibition of CaMKII and MEK1/2 results in attenuated ET_B_ receptor upregulation and improved neurological outcome after SAH [29]. The present study was designed to evaluate the time-dependent effects of CaMKII and MEK1/2 inhibitors on signaling kinases and proteins involved in cerebrovascular inflammatory responses using an *in vitro* approach that mimics several of the ischemic-like vascular wall changes [5,9].

The present results suggest an important role and cross-talk between CaMKII and ERK1/2 kinases in the inflammatory and apoptotic activity in the VSMCs. The novel finding of this study is that administration of a CaMKII inhibitor at 0 hours or a MEK1/2 inhibitor as late as 6 hours after initiating incubation has pronounced effects on inflammatory signals in the cerebral vessel walls, in particular in the smooth muscle cells. The study demonstrates the importance of specific time points in intracellular signaling, and that activation of inflammatory cascades at the beginning of incubation may not affect the inhibitory effect of the MEK1/2 antagonist U0126.

Levels of p-ERK1/2 and p-CaMKII were evaluated by western blot and immunohistochemistry. Previous studies have shown that p-CaMKII levels were highest at 0 hours [22] while p-ERK1/2 increased during the organ culture and peaked between 3 and 6 hours of incubation [21,30]. This study confirms our hypothesis that addition of a CaMKII inhibitor at 0 hours, or a MEK1/2 inhibitor at a time interval between 0 and 6 hours, has a strong inhibitory effect on the action of these kinases and their downstream inflammatory-associated targets. Although p-CaMKII expression was found to be reduced at 6 and 24 hours of incubation by the organ culture itself, treatment with KN93 at 0 hours may prevent any residual p-CaMKII from activating ERK1/2. It may also be that p-CaMKII acts on ERK1/2 in a negative feed-back mechanism, however these speculations need to be validated in further studies.

JNK and p38 are often associated with intracellular signaling related to inflammation. It has been shown that p-JNK and p-p38 increase in rat cerebral arteries during organ culture, ischemic stroke (MCAO) and SAH [7]. Here we examined the effects of CaMKII and MEK1/2 inhibitors on the phosphorylation of JNK and p38 induced by incubating arteries for 24 hours in the presence or absence of KN93 or U0126. In the present study, KN93 or U0126 significantly inhibited JNK and p38 activity, which could be an explanation for the decrease in inflammation in the vessel wall during organ culture. In support of this concept, KN93 was reported to inhibit MEK/ERK1/2 and JNK activation in platelet activating factor (PAF)-induced proinflammatory response in THP-1 cells [31].

The relation between inflammation and apoptosis and the role of caspase-3 has been well established in different cell types [32]. It has been shown that caspase-dependent MEKK1 cleavage results in JNK activation and apoptosis [13]. This study demonstrates that KN93 not only has an inhibitory effect on increased caspase-3 mRNA levels, but also on p-JNK and p-p38 during organ culture. This response was more pronounced when KN93 was added at the start of the incubation. In contrast, the MEK1/2 inhibitor decreased caspase-3 mRNA levels significantly when administered up to 6 hours after initiating the incubation. It seems that U0126 has its most potent effect when given at 6 hours. This is in agreement with our previous study that evaluated possible cross-talk between CaMKII and ERK1/2 in the process of endothelin receptor upregulation during organ culture [22]. We demonstrate here significantly attenuated ERK1/2 activity after 24 hours of organ culture when the CaMKII inhibitor KN93 was given at 0 or 6 hours, however the ERK1/2 inhibitor U0126 did not affect the CaMKII activity. Taken together, the previous and present studies suggest that upstream CaMKII activation results in the activation of ERK1/2 with subsequent downstream inflammatory and apoptotic consequences.

TNFR1 has an important role in apoptosis and inflammation within many cell types [33]. TNFRs can be activated by TNF-α directly [24], by serum starvation in human colon carcinoma cells [23], or found in human airway smooth muscle cells [34] which results in activation of JNK, p38, and ERK1/2 [34]. Interestingly, our study showed that U0126 decreased TNFR1 protein expression indicating that there is cross-talk between ERK1/2 and TNFR1 signaling. In support of this, a study has reported that U0126 attenuated TNFR1 expression in cerebral vessels after organ culture and ischemic stroke [5].

A relation between CaMKII and inflammatory molecules such as TNF-α, NF-κB [35], and T cell receptors have been previously reported [36]. Activation of NF-κB receptor activator or T cell receptor/CD3 leads to the activation of signaling cascades which includes calcium signaling and CaMKII [35,36]. The inhibitory effect of KN93 on TNFR1 suggests a possible cross-talk between CaMKII and TNFR1 in cerebrovascular inflammation during organ culture. Interestingly, U0126 and KN93 have inhibitory effects both on the upregulation of endothelin receptors and on inflammation in cerebral VSMCs. The present study suggests time-dependent involvement of CaMKII and ERK1/2 in cerebrovascular inflammation using an *in vitro* model that mimics ischemic-like vascular wall changes. Using the same *in vitro* model, a previous study of cerebral arteries has shown that TNF-α potentiates ET_B_ receptor-mediated contraction [37], thus linking inflammation with cerebrovascular receptor upregulation. On the other hand, TNF-α and IL-6 themselves may cause enhanced endothelin-1 production that can interact with ET_A_ and ET_B_ receptors in VSMCs and promote vasoconstriction [24]. We propose that there is involvement of an inflammatory mechanism that affects endothelin receptor upregulation in cerebral arteries. Further studies are needed to reveal the molecular relationship between receptor upregulation and inflammation, as well as validation of the present observations using *in vivo* stroke models, which eventually could uncover novel targets for stroke therapy.

## Conclusions

This study shows the importance of the timely activation of ERK1/2 and CaMKII signaling and the effect on downstream inflammatory-associated kinases and mediators. Inhibition of these signals at appropriate time points may have an important role in reducing cerebrovascular inflammation, and in turn, cerebral ischemic damage.

## Abbreviations

BSA: Bovine serum albumin; CAMK: Calcium-calmodulin dependent protein kinase; DMEM: Dulbecco´s modified Eagle´s medium; DMSO: Diemethyl sulfoxide; EF-1: Elongation factor; ERK1/2: Extracellular signal regulated kinase 1/2; ET-1: Endothelin 1; ET A B: Endothelin receptor A, B; GAPDH: glyceraldehyde 3-phosphate dehydrogenase; HRP: Horseradish peroxidase; IL-1: Interleukin-1; JNK: c-jun N-terminal kinase; MAPK: Mitogen activated protein kinase; MCA: Middle cerebral artery; MCAO: Middle cerebral artery occlusion; MEK/ERK 1/2: MAPK kinase/extracellular signal-regulated kinase1/2; mRNA: Messenger ribonucleic acid; NF-κB: Nuclear factor kappa B; PBS: Phosphate buffer-saline; PBST: Phosphate buffered-saline containing 0,25% Triton X-100; PCR: Polymerase chain reaction; SAH: Subarachnoid hemorrhage; SEM: Standard error of the mean; TNF-α: Tumor necrosis factor alpha; TNFR: Tumor necrosis factor receptor; TRIS-HCl: Trizma hydrochloride; T-TBS: Trizma buffered saline; VSMC: Vascular smooth muscle cell.

## Competing interests

The authors declare no competing interests.

## Authors’ contributions

RW conceived of the study, its design, performed the real-time PCR experiments, analyzed all data and drafted the manuscript. SE carried out the immunohistochemistry experiments and helped to draft the manuscript. HA performed parts of the immunohistochemistry experiments and helped to draft the manuscript. LEJ carried out the western blot and helped to draft the manuscript. LE participated in designing the study and drafted the manuscript. All authors have read and approved of the final version of the manuscript.
